# Effects of Selenium Application on Starch Structure, Physicochemical Properties, and Texture Properties of Rice

**DOI:** 10.1155/ijfo/6635000

**Published:** 2025-09-23

**Authors:** Xiaobin Tang, Rui Xu, Yong Sui, Shuyi Li, Zhenzhou Zhu, Fang Luo, Yuedi Huang, Jianbin Shi, Sha Cai, Tian Xiong, Fang Cai, Xin Mei

**Affiliations:** ^1^ Agro-Product Processing Research Sub-Center of Hubei Innovation Center of Agriculture Science and Technology, Wuhan, China; ^2^ School of Modern Industry for Selenium Science and Engineering, Wuhan Polytechnic University/National R&D Center for Se-Rich Agricultural Products Processing/Hubei Engineering Research Center for Deep Processing of Green Se-Rich Agricultural Products, Wuhan, China; ^3^ Key Laboratory of Agro-Products Cold Chain Logistics, Ministry of Agriculture and Rural Affairs, Institute of Agro-Products Processing and Nuclear-Agricultural Technology, Hubei Academy of Agricultural Science, Wuhan, China, agri.gov.cn

**Keywords:** eating quality, physicochemical properties, rice, selenium, starch

## Abstract

The consumption of selenium‐enriched rice represents an effective approach for human selenium intake. However, the potential effects of selenium on rice texture after cooking and the physicochemical properties of rice starch remain insufficiently understood. In this study, four selenium‐enriched rice varieties and one conventional rice variety were selected to compare their nutritional components and eating quality and to investigate the microstructure and physicochemical properties of their starches. The results showed that selenium content in selenium‐enriched rice was significantly higher than that in conventional rice (*p* < 0.01). Selenium enrichment had a positive effect on selenium accumulation, thereby promoting protein synthesis, with protein content increased by 3.01%–9.4%. However, it should be noted that the eating quality of selenium‐enriched rice was inferior to that of conventional rice, as indicated by lower taste values. Compared to conventional rice, the resistant starch content of selenium‐enriched rice increased by 13.41%–19.95%, the crystallinity increased by 4.89%–9.14%, the ordered double‐helical structure of starch granules was enhanced, and the gelatinization temperature increased. This study provides a theoretical reference for the processing, product development, and quality improvement of selenium‐enriched rice.

## 1. Introduction

As an essential trace element for the human body, selenium plays an irreplaceable role in physiological processes such as antioxidation, thyroid hormone metabolism, and immune regulation [1]. However, approximately one billion people worldwide suffer from selenium deficiency, and dietary selenium supplementation has become a crucial approach to safeguarding human health.

Rice, as a staple food for more than half of the world’s population, holds a significant position in the daily diet structure. A large number of studies have confirmed that foliar spraying of selenium fertilizers can increase the selenium content in rice [2], and consuming selenium‐enriched rice is an effective way to meet the human selenium nutritional requirements [3, 4]. Since agronomic measures during rice growth and development can affect the internal structure and physicochemical properties of rice starch [5], the addition of selenium is not merely a simple nutritional fortification process; instead, it can influence the internal structure and eating quality of rice. Starch, as the main component of rice (accounting for approximately 70%–80%) [6], its granule structure, crystallization characteristics, and gelatinization behavior directly determine the cooking and eating quality of rice. Existing research has shown that selenium mainly affects the metabolism of D‐amino acids, starch, sucrose, and linoleic acid in rice [7]. Selenium treatment may significantly change the starch structure by affecting the activity of enzymes related to starch synthesis and altering the ratio of amylose and amylopectin, thereby leading to fluctuations in eating quality indicators such as the stickiness, elasticity, and hardness of rice. Currently, the nutritional fortification function of selenium‐enriched rice has been widely recognized. Most existing studies focus on the correlation between selenium content and a single quality index, and there is a lack of systematic research on the effects of selenium on the eating quality of rice and the physicochemical properties of rice starch.

Therefore, this study is aimed at exploring the effects of selenium treatment on the nutritional components, eating quality, microstructure, and physicochemical properties of rice starch in rice. This study can provide a theoretical reference for the evaluation and improvement of the edible and nutritional quality of rice, thus contributing to the development of selenium‐enriched agricultural products.

## 2. Materials and Methods

### 2.1. Materials

Two Japonica rice varieties (Xiadao No. 1, Huaxiaxiangsi) and two Indica rice varieties (Nanjing 5055, Nanjing 9108) were provided by the Food Crops Institute, Hubei Academy of Agricultural Sciences (Wuhan, China). Se‐enriched rice in the treatment group was sprayed with 5000 mg/L selenium fertilizer at the filling stage in the field. The ordinary rice as the control group was sprayed with the same amount of water during the same period.

### 2.2. Difference in Eating Flavor Quality of Cooked Rice

The milled rice samples were cooked by an electric rice cooker as the previous method by Xu et al. [8]. When the cooked rice was cooled to room temperature, a rice taste analyzer (STA1B, SATAKE Co. Ltd., Japan) was used to measure the eating flavor quality.

### 2.3. Starch Isolation

The wet method was used to isolate rice starch from rice grains with minor modifications [9]. Rice flours (100 g) were soaked in a sodium hydroxide solution (0.3%, *w*/*w*) at room temperature for 24 h and then stirred for 1 h to remove proteins and lipids. A total of three washes with water and two with absolute ethanol were performed on the purified starch. A repeat of this treatment was performed until the water layer became clear, and the starch was oven‐dried at 40°C overnight. For further use, rice starch was ground and sieved through a 100‐mesh sieve.

### 2.4. The Main Nutritional Components of Rice

The rice samples of each treatment were pulverized into rice flour with three repetitions. The amylose contents of rice flour were determined as described by Shi et al. [10]. The total selenium content was determined by using inductively coupled plasma mass spectrometry (ICP‐MS 7900, Agilent Technologies Inc., United States). The protein content was calculated from the total nitrogen content using the Kjeldahl method with a conversion coefficient of 5.95 [11]. Ash content was determined using the experimental muffle furnace. Lipid content was determined using Soxhlet extraction (petroleum ether was used to extract lipids).

### 2.5. Granule Size Distribution

Starch granule size distribution was analyzed using a mastersizer (Malvern Mastersizer 2000, Malvern Instruments Ltd., United Kingdom) at the range of 0.1–3000 *μ*m.

### 2.6. Scanning Electron Microscopy

A scanning electron microscope was used to examine the morphological characteristics of rice starch (Nova Nano SEM 450, FEI Company, Holland). Double‐sided tape was used to secure the dried starch sample to the loading platform, and gold was sputtered on it. Then, the samples were observed at 5 kV and 1000× magnification.

### 2.7. X‐Ray Diffraction Analysis

XRD patterns of rice flour were prepared using an x‐ray diffractometer (x’pert3 powder, PANalytical B.V. Netherlands) equipped with Cu‐K*α* radiation. Measurements were obtained at room temperature with a scanning rate of 0.013°/s and a diffraction angle range of 5°–50° (2‐theta° range), where theta is the angle of incidence of the x‐ray beam on the sample. The diffraction patterns were analyzed using MDI Jade 6 software. Relative crystallinity was calculated by the ratio of the crystalline area to the total diffractogram area [12]:

Crystallinity%=AcAc+Aa×100%

where *A*_c_ is the area of crystalline and *A*_a_ is the area of amorphous phases.

### 2.8. Fourier Transform Infrared Spectroscopy (FTIR)

The rice starch was dried, ground with potassium bromide powder at a ratio of 1:100, and compressed into thin tablets. A Fourier transform infrared spectrometer (Nicolet iS50R, Thermo Scientific Corporation, United States) was used to scan the samples. The scanning wavelength range was 400–4000 cm^−1^, the number of scans was 32, and the resolution was 4 cm^−1^ [13].

### 2.9. Raman Spectroscopy

Raman spectra of the rice starches were obtained using a Laser Confocal Raman Spectrometer (LabRAM HR800, Horiba Jobin Yvon Corporation, France). The laser wavelength was 532 nm, and the laser power and exposure time were 25 mW and 15 s, respectively. Approximately 10 mg of each sample was used for Raman spectroscopy analysis.

### 2.10. Pasting Properties

The gelatinization characteristics of rice starch were measured by Rapid Visco Analyzer (Super RVA 4, Newport Scientific, Australia). The pasting characteristic parameters such as gelatinization temperature (GT), peak viscosity (PV), trough viscosity (TV), and final viscosity (FV) were recorded, and the breakdown (BD) and setback (SB) were calculated according to the gelatinization characteristic curve of the sample [14].

### 2.11. In Vitro Starch Digestibility Properties

In vitro digestibility of the rice starches was tested according to the method of Ma et al. [15]. The starch sample was suspended in sodium acetate buffer and heated for 30 min at 95°C. After gelatinization, samples were treated with an enzyme mixture (pancreatic *α*‐amylase and amyloglucosidase) at 37°C. To calculate the rapidly digestible starch (RDS, digested within 20 min), slowly digestible starch (SDS), and resistant starch (RS undigestible starch after 120 min), glucose levels were determined in the enzymatic hydrolysates after 0, 20, and 120 min of hydrolysis:

RDS%=G20−G0TS×0.9100×SDS%=G120−G20TS×0.9100×RS%=TS−RDS−SDSTS×100

where *G*_0_, *G*_20_, and *G*_120_ are the contents of glucose released within 0, 20, and 120 min of hydrolysis, respectively; TS is total starch weight.

### 2.12. Statistical Analysis

Each experiment was repeated three times, and all data were expressed as mean and standard deviation. The statistical analysis was performed by ANOVA using SPSS 26 (SPSS Inc., Chicago, Illinois, United States). The mean values obtained were compared by Duncan test (*p* < 0.05). The graphs were processed by Origin (Version 2021, Microcal Inc., Northampton, Massachusetts, United States).

## 3. Results and Discussion

### 3.1. The Main Nutritional Components

As shown in Table 1, the total selenium content of selenium‐rich rice was significantly higher than that of ordinary rice (*p* < 0.01). The protein content of Se‐enriched rice was slightly higher than that of ordinary rice among the same varieties, and the Se‐enriched rice of Huaxiaxiangsi showed a significant difference (*p* < 0.05). Previous studies have shown that selenium replaces sulfur along the absorption and metabolism pathway of sulfur and combines with cysteine and methionine in proteins to form seleno‐amino acid compounds, which are further transported to rice grains and stored in proteins in the form of selenoprotein, resulting in significant differences in protein content [16]. Hu et al. [3] found that spraying selenium fertilizer could increase grain protein content, decrease cysteine content, and increase the methionine content of rice. As for the ash content of rice, the selenium‐enriched treatment makes the ash content of rice higher than that of ordinary rice. This is because the selenium fertilizer sprayed on the leaf surface of rice during the filling stage is partly stored in the body by the rice grains in the form of inorganic selenium [17]. The content of amylose was between 11% and 15%, and there was no significant difference between Se‐enriched rice and ordinary rice (*p* > 0.05). For crude fat of rice, the results showed that the crude fat content of Huaxiaxiangsi and Nanjing 9108 control group was more than 1%, and the crude fat content of other rice samples was less than 1%. There was no significant difference between Se‐enriched rice and ordinary rice (*p* > 0.05).

**Table 1:** The main nutritional components of the Se‐enriched rice and ordinary rice.

**Crop type**		**Treatments**	**Amylose content (%)**	**Total selenium (%)**	**Protein content (%)**	**Lipid content (%)**	**Ash (%)**
Japonica	XD	Se‐R	13.41 ± 0.19	0.46 ± 0.01^∗∗^	7.18 ± 0.46	0.86 ± 0.03	0.51 ± 0.04
		OR	13.10 ± 0.18	0.02 ± 0.01	6.97 ± 0.36	0.82 ± 0.03	0.49 ± 0.06
	HX	Se‐R	14.09 ± 0.13^∗^	0.32 ± 0.04^∗∗^	6.98 ± 0.03^∗^	0.91 ± 0.10	0.47 ± 0.02
		OR	13.27 ± 0.12	0.02 ± 0.03	6.38 ± 0.27	1.01 ± 0.01	0.45 ± 0.15

Indica	N.5	Se‐R	11.34 ± 0.22^∗^	0.41 ± 0.02^∗∗^	7.56 ± 0.10^∗^	0.88 ± 0.01	0.50 ± 0.01^∗^
		OR	12.12 ± 0.16	0.02 ± 0.01	6.99 ± 0.17	0.84 ± 0.07	0.41 ± 0.02
	N.9	Se‐R	12.46 ± 0.12^∗^	0.46 ± 0.02^∗∗^	7.48 ± 0.02	0.97 ± 0.02^∗^	0.52 ± 0.03^∗^
		OR	12.89 ± 0.16	0.03 ± 0.01	7.15 ± 0.65	1.13 ± 0.08	0.43 ± 0.01

*Note:* Results were given in dry matter.

Abbreviations: HX, Huaxiaxiangsi; N.5, Nanjing 5055; N.9, Nanjing 9108; OR, ordinary rice; Se‐R, Se‐enriched rice; XD, Xiadao No. 1.

^∗^Significant difference between Se‐enriched rice and ordinary rice of the same cultivar at the level of *p* = 0.05.  ^∗∗^Significant difference between Se‐enriched rice and ordinary rice of the same cultivar at the level of *p* = 0.01.

### 3.2. Difference in Eating Flavor Quality of Cooked Rice

According to the principle of neural fuzziness, the rice tasting instrument establishes the relationship between the physicochemical index content (amylose, protein, moisture, etc.) and the taste of rice using near‐infrared microlight transmission and then makes a comparison with the attached standard rice to obtain the taste value [18]. Compared to conventional rice, selenium‐enriched rice exhibited slightly lower appearance quality and a 2.84%–10.05% reduction in taste value. In terms of texture, selenium‐enriched rice showed significantly higher hardness and lower viscosity compared to conventional rice, while the effect on elasticity was not significant. For instance, the elasticity value of selenium‐enriched rice was 0.96 for japonica variety XD compared to 0.95 for conventional rice; for indica variety N.5, selenium‐enriched rice recorded 0.84 compared to 0.86 for conventional rice. Notably, selenium enrichment significantly reduced the taste value (*p* < 0.01). Previous studies have indicated that viscosity, flavor, and appearance are positively correlated with eating quality, and all these indices were lower in selenium‐enriched rice compared to conventional rice, suggesting that selenium enrichment reduced the eating quality of rice [19]. The taste value is positively related to the eating quality of rice. The results of eating quality determination of Se‐enriched rice and ordinary rice are shown in Table 2. There were significant differences in taste between selenium‐rich rice and ordinary rice (*p* < 0.05), and the taste value of selenium‐rich rice was generally lower than that of ordinary rice. The appearance quality of rice varieties was also slightly reduced by selenium‐enriched treatment. In terms of hardness and viscosity, the hardness and viscosity of selenium‐rich rice were higher than those of ordinary rice, and the hardness and viscosity of XD and N.5 were significantly different (*p* < 0.05). However, selenium‐enriched treatment increased the elasticity of two japonica rice (XD and HX) and decreased the elasticity of two indica rice (N.5 and N.9). It is worth noting that selenium‐enriched treatment significantly decreased the taste value of rice (*p* < 0.01), which may be the result of the comprehensive effect of selenium‐enriched treatment on the appearance, taste, and spring of rice.

**Table 2:** The eating quality of Se‐enriched rice and ordinary rice.

**Crop type**		**Treatments**	**Appearance**	**Taste**	**Hardness**	**Stickiness**	**Spring**	**Taste value**
Japonica	XD	Se‐R	5.57 ± 0.06^∗^	5.37 ± 0.06^∗^	0.65 ± 0.02^∗^	0.06 ± 0.01^∗^	0.96 ± 0.02	63.67 ± 0.58^∗∗^
		OR	6.30 ± 0.10	5.93 ± 0.12	0.33 ± 0.03	0.11 ± 0.02	0.95 ± 0.03	66.67 ± 0.58
	HX	Se‐R	7.07 ± 0.06^∗^	6.50 ± 0.01^∗^	0.31 ± 0.03	0.16 ± 0.01	0.94 ± 0.05	72.00 ± 0.01^∗∗^
		OR	7.30 ± 0.01	6.90 ± 0.01	0.28 ± 0.03	0.17 ± 0.01	0.93 ± 0.05	75.00 ± 0.01

Indica	N.5	Se‐R	5.27 ± 0.06	5.37 ± 0.06^∗^	0.13 ± 0.02^∗^	0.03 ± 0.01^∗^	0.84 ± 0.01	62.67 ± 0.58^∗∗^
		OR	6.50 ± 0.10^∗^	6.40 ± 0.10	0.27 ± 0.02	0.14 ± 0.01	0.86 ± 0.01	69.67 ± 0.58
	N.9	Se‐R	5.63 ± 0.06	5.07 ± 0.06^∗^	0.08 ± 0.01	0.06 ± 0.01	0.92 ± 0.01	68.33 ± 0.58^∗∗^
		OR	5.73 ± 0.06	5.27 ± 0.06	0.12 ± 0.01	0.08 ± 0.01	0.96 ± 0.01	70.33 ± 1.15

Abbreviations: HX, Huaxiaxiangsi; N.5, Nanjing 5055; N.9, Nanjing 9108; OR, ordinary rice; Se‐R, Se‐enriched rice; XD, Xiadao No. 1.

^∗^Significant difference between Se‐enriched rice and ordinary rice of the same cultivar at the level of *p* = 0.05.  ^∗∗^Significant difference between Se‐enriched rice and ordinary rice of the same cultivar at the level of *p* = 0.01.

### 3.3. Granule Size Distribution and Granule Morphology of Rice Starch

**Figure 1:**
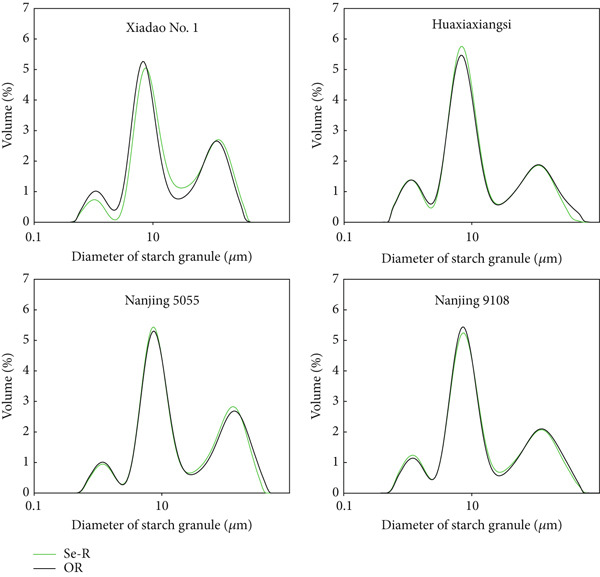
Starch granule volume distribution for the Se‐enriched rice starch and ordinary rice starch. Se‐R: Se‐enriched rice starch; OR: ordinary rice starch.

**Figure 2:**
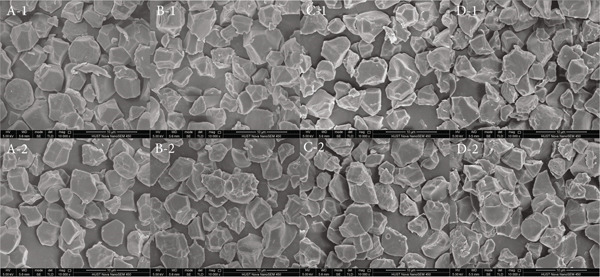
Microstructural morphology of Se‐enriched rice starch and ordinary rice starch. A: Xiadao No. 1; B: Huaxiaxiangsi; C: Nanjing 5055; D: Nanjing 9108. 1: Se‐enriched rice starch; 2: ordinary rice starch.

The particle size distribution curves of starches from selenium‐enriched and conventional rice are shown in Figure 1. It can be observed that selenium treatment had no significant effect on starch particle size distribution, with all samples exhibiting three distinct peaks. This may be attributed to the stepwise formation of starch granules during biosynthesis, where small primary granules are initially formed, followed by molecular aggregation and growth leading to medium and large granules [20], from which it can also be seen that the particle size of 1–10 *μ*m in all starch samples accounts for the highest proportion. SEM images of starch isolated from Se‐enriched rice and ordinary rice are shown in Figure 2. For all four starches, granules exhibited typical rice starch morphology of polyhedral shape. Based on previous rice starch research, this observation was confirmed [21, 22]. There was a small difference in the particle morphology of Se‐enriched rice starch and ordinary rice, which was consistent with the particle size analysis results.

### 3.4. Measurement of the X‐Ray Diffraction Pattern

**Figure 3:**
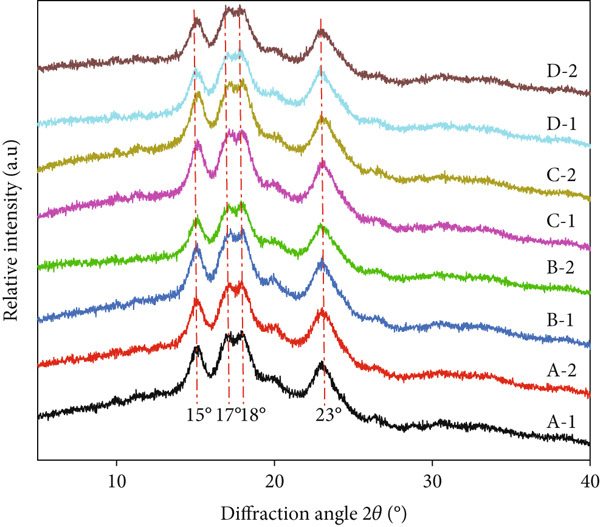
Comparison and difference between the x‐ray powder diffraction patterns between the Se‐enriched rice and ordinary rice. A: Xiadao No. 1; B: Huaxiaxiangsi; C: Nanjing 5055; D: Nanjing 9108. 1: Se‐enriched rice starch; 2: ordinary rice starch.

As shown in Figure 3, the diffraction pattern of rice starch was not altered, and it showed typical A‐type crystal characteristics with strong diffraction peaks around 15°, 17°, 18.0°, and 23° at 2*θ* and weak diffraction peaks around 20° [23]. No new diffraction peak was observed, indicating that the Se‐enriched treatment did not change the crystal form of rice starch. However, the crystallinity of several Se‐enriched rice starch samples was 30.59%, 38.32%, 28.98%, and 25.48%, respectively, from which we could observe that the Se‐enriched treatment led to an increase in starch crystallinity (Table 3). Because fertilization patterns were different when both Se‐enriched crops and ordinary crops were grown in the same fields, the crystalline structure of the starches may also be affected [13]. It seems that the rice starches from Se‐enriched rice had a strong crystalline structure, possibly due to the Se‐enriched treatment during grain filling.

**Table 3:** The crystallinity, R1047/1022, R1022/995, and FWHM of Se‐enriched rice starch and ordinary rice starch.

**Crop type**		**Treatments**	**Crystallinity (%)**	**R1047/1022**	**R1022/995**	**FWHM**
Japonica	XD	Se‐R	30.59 ± 0.36^∗^	1.20 ± 0.01^∗^	1.04 ± 0.01^∗^	17.63 ± 0.21
		OR	28.36 ± 0.12	1.11 ± 0.02	1.29 ± 0.01	17.89 ± 0.11
	HX	Se‐R	38.32 ± 0.10^∗^	1.16 ± 0.01	1.18 ± 0.02^∗^	16.36 ± 0.12^∗^
		OR	35.11 ± 0.56	1.12 ± 0.03	1.25 ± 0.02	16.78 ± 0.11

Indica	N.5	Se‐R	28.98 ± 0.24^∗^	1.26 ± 0.01^∗^	1.11 ± 0.01^∗^	17.25 ± 0.12^∗^
		OR	27.63 ± 0.46	1.22 ± 0.01	1.22 ± 0.04	18.15 ± 0.11
	N.9	Se‐R	25.48 ± 0.26^∗^	1.31 ± 0.02	1.08 ± 0.02	15.41 ± 0.08^∗^
		OR	23.76 ± 0.06	1.29 ± 0.01	1.12 ± 0.01	16.26 ± 0.16

Abbreviations: HX, Huaxiaxiangsi; N.5, Nanjing 5055; N.9, Nanjing 9108; OR, ordinary rice; Se‐R, Se‐enriched rice; XD, Xiadao No. 1.

^∗^Significant difference between Se‐enriched rice and ordinary rice of the same cultivar at the level of *p* = 0.05.

### 3.5. The Fine Structure of Rice Starch

FTIR is widely acknowledged for its sensitivity to the short‐range order, specifically the double‐helix composition in starch. This property has been effectively utilized in the examination of starch granule crystallinity and the amorphous regions adjacent to the granule surface. The infrared absorption spectrum could reflect the changes and vibration of the groups in the starch molecule [24]. The infrared spectra of starch granules from different sources are similar in shape but different in intensity (Figure 4a). All samples had a strong and wide peak near 3200–3650 cm^−1^. The FTIR intensity ratios at 995, 1022, and 1047 cm^−1^ indicate the proportion of ordered to amorphous starch, and it is related to the amorphous and crystalline structures of starch [25]. The 1047/1022 and 1022/995 cm^−1^ ratio from the deconvoluted FTIR spectrum can therefore be used as a convenient index of FTIR compared to other measurements of starch conformation [26]. The FTIR spectra of starch were similar among the four rice starch samples. However, on the basis of the calculation of relative intensities of FTIR bands at 1045, 1022, and 995 cm^−1^ from the baseline to peak height, we determined that the starch from Se‐enriched rice tended to have higher 1047/1022 and 1022/995 cm^−1^ ratios (Table 3). These results suggest that the double helical ordered structure of starch granules from Se‐enriched rice was increased, which is in agreement with the data from XRD analysis.

Figure 4FTIR and Raman spectra of Se‐enriched rice starch and ordinary rice starch. (a) FTIR spectrum; (b) Raman spectrum. A: Xiadao No. 1; B: Huaxiaxiangsi; C: Nanjing 5055; D: Nanjing 9108. 1: Se‐enriched rice starch; 2: ordinary rice starch.

**Figure:**
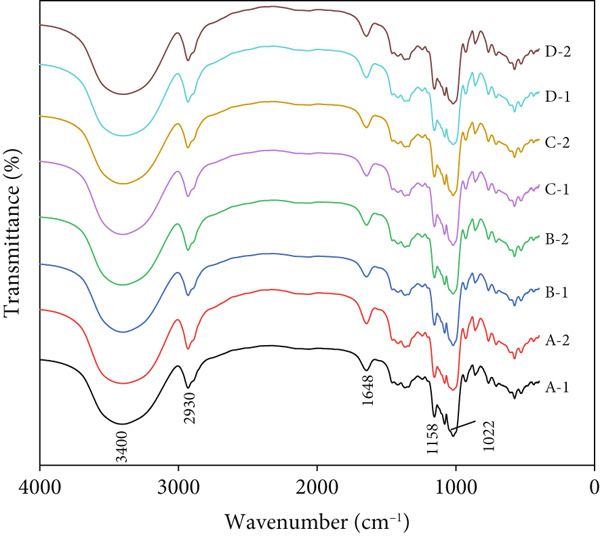
(a)

**Figure:**
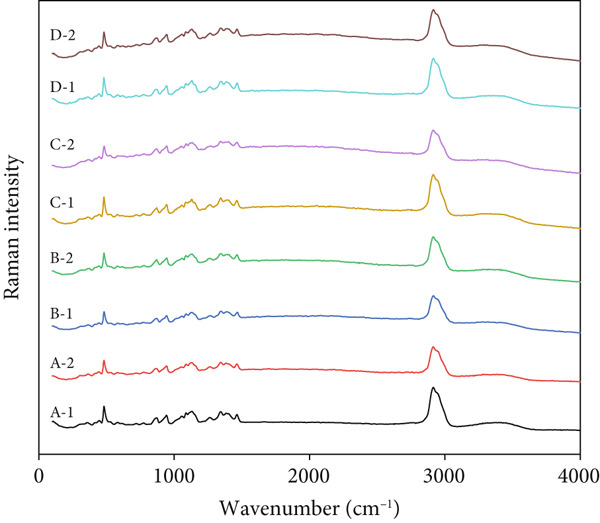
(b)

Based on previous reports, the helical structures of starch are related to the characteristic peaks of the Raman spectra [27, 28]. The Raman spectra of the starch samples are presented in Figure 4b. All spectra of starches obtained from Se‐enriched rice and ordinary rice are highly similar, from which all of the principal characteristic bands for starch are observed, and it is visible that the positions of the observed bands are almost the same. In addition, no new characteristic peaks appeared in the Raman spectra of Se‐enriched rice starch, indicating that there was no covalent interaction between starch and selenium. Furthermore, the full width at half maximum (FWHM) of the Raman band at 480 cm^−1^ has been widely used to measure and compare the structural change of starch samples. The lower the FWHM, the higher the molecular order of starch [29]. According to the calculation results in Table 3, it can be seen that the FWHM of Se‐enriched rice starch is lower than that of ordinary rice starch, indicating that although the selenium‐rich treatment does not produce a new covalent effect, it improves the molecular order of starch.

### 3.6. The Pasting Properties

**Figure 5:**
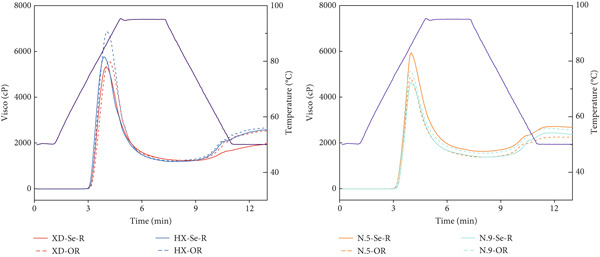
RVA spectra of Se‐enriched rice starch and ordinary rice starch. XD: Xiadao No. 1; HX: Huaxiaxiangsi; N.5: Nanjing 5055; N.9: Nanjing 9108. Se‐R represents Se‐enriched rice; OR represents ordinary rice.

In sufficient water, RVA measurements reveal how the apparent viscosity of the sample changes with heating and cooling, which predicts the texture of rice starch [30]. As shown in Figure 5, the viscosity of starch from Se‐enriched rice and ordinary rice was different, but similar patterns of apparent viscosity change were observed. During the heating process, with the increase in temperature, the viscosity also began to increase. After the viscosity increases to a maximum value, it begins to decrease with increasing temperature. During cooling, the viscosity increases with decreasing temperature. This is because the starch solution is heated, the hydrogen bond strength between the particles is weakened, and the starch particles absorb water and expand, so the viscosity increases until reaching the PV. With continued heat, the starch particles continue to expand to rupture, resulting in amylose dissolution and viscosity decrease. During the cooling process, as the temperature decreases, amylose is retrograded, during which hydrogen bonds are formed again and the viscosity increases [31]. Six major parameters of the starch pasting curve, PV, TV, breakdown viscosity (BDV, PV minus TRV), FV, setback viscosity (SBV, FV minus TRV), and pasting temperature (PT), are shown in Table 4. Selenium treatment had significant effects on the gelatinization properties of rice starch, mainly reflected by reductions in PV, TV, and BD value, while increasing GT. No clear trends were observed for FV and SB value. According to Hu et al. [32], higher BD values are associated with larger water contact areas and easier disintegration of rice grains during cooking, contributing to better taste quality. The reduced BD values of selenium‐enriched rice further support the observation of its inferior eating quality compared to conventional rice. Overall, selenium treatment may enhance the thermal stability of starch by altering its ordered structure [33].

**Table 4:** RVA parameters of Se‐enriched rice starch and ordinary rice starch.

**Crop type**		**Variety**	**PV (cP)**	**TRV (cP)**	**BDV (cP)**	**FV (cP)**	**SBV (cP)**	**PT (°C)**
Japonica	XD	Se‐R	5354.00 ± 20.90	1187.50 ± 50.20	4166.50 ± 59.10^∗^	2821.50 ± 20.51	1634.00 ± 70.71^∗^	74.40 ± 0.57
		OR	5915.50 ± 28.99	1315.50 ± 51.62	4600.00 ± 22.63	2643.50 ± 13.44	1328.00 ± 65.05	74.00 ± 0.01
	HX	Se‐R	5775.50 ± 58.69^∗∗^	1177.00 ± 29.70	4598.50 ± 88.39^∗^	2568.50 ± 30.41	1391.50 ± 60.10	74.33.±0.60
		OR	6851.50 ± 81.32	1212.00 ± 9.90	5639.50 ± 71.42	2640.50 ± 99.70	1428.50 ± 109.60	73.58 ± 0.50

Indica	N.5	Se‐R	5934.50 ± 28.21^∗∗^	1620.00 ± 63.64^∗^	4314.50 ± 28.50	2672.50 ± 111.02	1052.50 ± 47.38^∗^	75.98 ± 0.04^∗^
		OR	4867.00 ± 78.41	1368.50 ± 105.36	3498.50 ± 73.05	2239.50 ± 54.86	871.00 ± 49.50	74.83 ± 0.53
	N.9	Se‐R	4643.50 ± 36.06	1372.50 ± 23.33^∗^	3271.00 ± 59.40	2362.50 ± 57.28	990.00 ± 33.94	75.60 ± 0.07^∗^
		OR	5119.50 ± 73.65	1536.50 ± 58.69	3583.00 ± 24.96	2552.50 ± 51.62	1016.00 ± 70.07	74.78 ± 0.04

Abbreviations: HX, Huaxiaxiangsi; N.5, Nanjing 5055; N.9, Nanjing 9108; OR, ordinary rice; Se‐R, Se‐enriched rice; XD, Xiadao No. 1.

^∗^Significant difference between Se‐enriched rice and ordinary rice of the same cultivar at the level of *p* = 0.05.  ^∗∗^Significant difference between Se‐enriched rice and ordinary rice of the same cultivar at the level of *p* = 0.01.

### 3.7. In Vitro Starch Digestibility Properties

Starch can be classified into three categories based on its in vitro digestibility: RDS, SDS, and RS [34]. It was suggested that the content of RS and SDS is responsible for the antidigestibility of rice starch. The amounts of these three components in the samples are shown in Table 5. Selenium‐enriched treatment reduced the content of RDS in rice starch and showed a significant difference in N.5. In general, the content of RS in selenium‐rich rice starch was higher than that in ordinary rice starch and showed a significant difference in XD and N.5. This may be related to the improvement of molecular order and higher crystallinity of Se‐enriched rice starch, resulting in higher thermal stability and digestive resistance [35, 36].

**Table 5:** The in vitro digestion of Se‐enriched rice and ordinary rice starch.

**Crop type**		**Treatments**	**RDS (%)**	**SDS (%)**	**RS (%)**
Japonica	XD	Se‐R	59.87 ± 0.36	34.90 ± 0.16	5.23 ± 0.09^∗^
		OR	60.31 ± 0.26	35.33 ± 0.22	4.36 ± 0.16
	HX	Se‐R	66.43 ± 0.45	29.68 ± 0.12	3.89 ± 0.26
		OR	67.30 ± 0.54	29.27 ± 0.15	3.43 ± 0.09

Indica	N.5	Se‐R	55.23 ± 0.49^∗^	41.21 ± 0.66^∗^	3.56 ± 0.06^∗^
		OR	59.26 ± 0.63	37.66 ± 0.54	3.08 ± 0.08
	N.9	Se‐R	51.56 ± 0.16	45.88 ± 0.44	2.56 ± 0.16
		OR	52.33 ± 0.36	45.49 ± 0.15	2.18 ± 0.15

Abbreviations: HX, Huaxiaxiangsi; N.5, Nanjing 5055; N.9, Nanjing 9108; OR, ordinary rice; Se‐R, Se‐enriched rice; XD, Xiadao No. 1.

^∗^Significant difference between Se‐enriched rice and ordinary rice of the same cultivar at the level of *p* = 0.05.

## 4. Conclusion

The selenium content and protein content of Se‐enriched rice were significantly increased, which made the nutritional quality of Se‐enriched rice better than that of ordinary rice. However, the Se‐enriched treatment gave the rice a lower taste quality. Although the appearance of rice starch did not change due to Se‐enriched treatment, the results of x‐ray diffraction, FTIR, and Raman spectroscopy showed that Se‐enriched treatment improved the crystallinity of rice starch and increased the ordered structure of starch, which made the GT of Se‐enriched rice higher than that of ordinary rice in the control group. The increased starch resistance in selenium‐enriched rice may be due to the improvement of starch crystallinity and molecular order, which enhances the thermal stability and resistance to digestion of starch. This study provided a theoretical reference for the processing and product development of Se‐enriched rice and the improvement of rice taste quality.

## Conflicts of Interest

The authors declare no conflicts of interest.

## Author Contributions

Xiaobin Tang and Rui Xu: data curation, writing—original draft. Yong Sui: supervision, reading of draft and final manuscript, funding acquisition. Shuyi Li: methodology, formal analysis, data curation. Zhenzhou Zhu: supervision, reading of draft and final manuscript. Fang Luo and Yuedi Huang: formal analysis. Jianbin Shi and Sha Cai: writing—review and editing. Tian Xiong: conceptualization, data curation. Fang Cai: writing—review and editing. Xin Mei: supervision, reading of draft and final manuscript, funding acquisition.

## Funding

This work was supported by the project of Special Project for Science and Technology Innovation of Wuhan (2022020801020344), the National Key Research and Development Program of China (10.13039/501100012166) (2018YFD0301306‐4‐2), the Outstanding Young and Middle‐Aged Scientific Innovation Team of Colleges and Universities of Hubei Province (10.13039/501100019076) (T2020012) and the Science and Technology Plan Project in Agriculture and Rural Areas of Hubei Province in 2023.

## Data Availability

Data are available on request from the authors.
